# Global Chromosomal Structural Instability in a Subpopulation of Starving *Escherichia coli* Cells

**DOI:** 10.1371/journal.pgen.1002223

**Published:** 2011-08-25

**Authors:** Dongxu Lin, Ian B. Gibson, Jessica M. Moore, P. C. Thornton, Suzanne M. Leal, P. J. Hastings

**Affiliations:** 1 Department of Molecular and Human Genetics, Baylor College of Medicine, Houston, Texas, United States of America; 2 Department of Biochemistry and Molecular Biology, Baylor College of Medicine, Houston, Texas, United States of America; Université Paris Descartes, INSERM U1001, France

## Abstract

Copy-number variations (CNVs) constitute very common differences between individual humans and possibly all genomes and may therefore be important fuel for evolution, yet how they form remains elusive. In starving *Escherichia coli*, gene amplification is induced by stress, controlled by the general stress response. Amplification has been detected only encompassing genes that confer a growth advantage when amplified. We studied the structure of stress-induced gene amplification in starving cells in the Lac assay in *Escherichia coli* by array comparative genomic hybridization (aCGH), with polymerase chain reaction (pcr) and DNA sequencing to establish the structures generated. About 10% of 300 amplified isolates carried other chromosomal structural change in addition to amplification. Most of these were inversions and duplications associated with the amplification event. This complexity supports a mechanism similar to that seen in human non-recurrent copy number variants. We interpret these complex events in terms of repeated template switching during DNA replication. Importantly, we found a significant occurrence (6 out of 300) of chromosomal structural changes that were apparently not involved in the amplification event. These secondary changes were absent from 240 samples derived from starved cells not carrying amplification, suggesting that amplification happens in a differentiated subpopulation of stressed cells licensed for global chromosomal structural change and genomic instability. These data imply that chromosomal structural changes occur in bursts or showers of instability that may have the potential to drive rapid evolution.

## Author Summary

Much of the difference between individual humans is in the number of copies of genes and lengths of genome. The mechanisms by which copy number variation arises are not well understood. We sought information on copy number change mechanisms by extensive use of array comparative genomic hybridization of whole genomes in bacteria selected for amplification of part of the genome. We report that about 10% of amplified isolates carried other chromosomal structural changes associated with the amplification, a result comparable to that seen in human copy number variants. Importantly, we found a significant occurrence of structural changes that were not involved in the amplification event. These were not seen in a control sample of stressed cells not carrying amplification. This establishes that chromosomal structural change happens in a subpopulation of cells apparently licensed to undergo these changes. Because the changes occur under the stress of starvation and require two of the cells' stress-response systems, we propose that licensing for cell-wide structural change in this subpopulation is a component of response to stress. This idea has implications for the mechanisms of evolution and cancer progression, suggesting that changes occur in a shower of events rather than as isolated random events.

## Introduction

Copy number variations (CNVs) are regions of DNA either deleted or duplicated/amplified relative to a reference genome. CNVs constitute the most ubiquitous differences between individual or personal human genomes [1], can be associated with many Mendelian and complex human diseases [2] because *de novo* events cause a significant fraction of sporadic birth defects [3] and are responsible for the selected rapid evolutionary changes accompanying animal domestication (e.g. [4]). In human, CNV arises either through non-allelic crossing-over between repeated sequences, giving recurrent end-points, or at non-recurrent positions. Non-recurrent events show two conspicuous features: many of them show complexity [5], often in the form of lengths of nearby sequence inserted at the novel junction, and second, the junctions tend to show microhomology of a few base-pairs, not sufficient to allow homologous recombination to occur (reviewed by [6], [7]). We and others have reported similar properties in our studies of amplification in *Escherichia coli*, namely that some of the events are complex, and the junctions show microhomology at the site of the joint making *E. coli* a useful model for studying the mechanisms that underlie human CNV [8], [9], [10].

Amplification at *lac* in the Lac assay system on an F′-plasmid in *E. coli* requires DNA polymerase I (Pol I) but not excision repair (also involving Pol I), placing the event at replication forks [10], [11]. Parenthetically, in yeast both break-induced replication (BIR) [12] and CNV [13] require the non-essential DNA polymerase subunit pol32. Furthermore, in *E. coli* amplification is enhanced by 3′ single-stranded DNA ends, suggesting priming of DNA synthesis [10]. Based on these observations we proposed the long-distance template-switch model, in which the 3′ primer-end at a stalled replication fork switches template to a different replication fork and anneals at a site of microhomology [10]. Repeated switches would explain the complexity at the junctions, and a template switch to a region already replicated would produce a duplication that could be expanded into amplification by unequal crossing-over. However, amplification also requires TraI [14], an endonuclease that nicks the F-plasmid at the origin of transfer, *oriT*, and this requirement is suppressed by double-strand cutting near *lac* on the F′-plasmid [14]. Taking these findings together with the report that BIR repair of collapsed (broken) replication forks in yeast shows frequent template switching [15], we proposed that microhomology-mediated (MM) events might occur by a modification of BIR (MMBIR) whereby repair is achieved by annealing of the 3′-tail at a collapsed fork with any nearby single-stranded DNA [6]. Annealing would have lower homology requirements than homologous recombination, and hence explain the microhomology junctions. Another possible explanation for recombination at sites of microhomology is non-homologous end-joining (NHEJ). NHEJ requires two double-strand breaks to make every heterologous junction, and consequently complex events would require multiple DNA double-strand breaks. NHEJ fails to explain the requirement for DNA polymerase I or the involvement of 3′ DNA ends in amplification. For these reasons we do not favor NHEJ as a mechanism for adaptive amplification in the Lac assay, nor is it our preferred mechanism to explain microhomology observed at human genomic deletion rearrangements with a single junction; the latter being explained more parsimoniously by a single template switch [1], [5].

In the Lac assay in *E. coli* [16], stationary phase Lac^−^ cells carrying a +1 frameshift mutation are spread on lactose minimal medium. Lac^+^ colonies arise over days from the starving cells. The colonies carry either amplified arrays of the leaky *lac* allele or a compensating frame-shift mutation (point mutants) [17]. The point-mutant Lac^+^ colonies are found to carry secondary unselected mutations at a high frequency (up to 10^−2^ for some loci) [18], [19], [20]. Starved cells on the same plate that did not mutate to Lac^+^ carry a much lower frequency of unselected mutations [19]. Thus, some or all Lac^+^ colonies arise from a hypermutating subpopulation (HMS) while the majority of the starved cells do not take part in hypermutability. The HMS is defined by the stress responses that are activated in given cell [21], [22]. It has not been established whether or not amplified Lac^+^ colonies arise from a chromosomally unstable subpopulation, though it has been shown that they do not arise from the HMS [17].

This study reports the use of array comparative genomic hybridization (aCGH) to analyze genome-wide changes in copy number. We sought, first, evidence of secondary unselected cell-wide chromosomal structural instability in those cells that carry amplification at *lac*. Evidence of secondary chromosomal structural change in amplified isolates that is not seen in controls constitutes evidence of a physiological difference that affects genome stability between cells undergoing amplification and those that do not. We found a significantly higher occurrence of unselected events that would not have bestowed a growth advantage among amplified isolates compared with stressed Lac^−^ control cells. This demonstrates that amplification is happening in a differentiated subpopulation undergoing general chromosomal structural change, suggesting that this differentiation might be mediated by stress responses. Second, we sought further evidence that amplification in *E. coli* shows similar complexity to human non-recurrent CNV events. We found complexity in the amplification events in over 7% of amplified isolates, mostly in the form of inverted duplications within the amplicons (units of amplification), confirming that there is a tendency for events that mediate chromosomal structural change to be complex.

## Results

Using Oxford Gene Technologies 44K aCGH arrays, giving a resolution of about 100 base-pairs (bp), we analyzed 300 isolates that had an unstable Lac^+^ phenotype, in comparison with a reference DNA sample from the parental strain FC40 [16]. The 300 unstable Lac^+^ isolates consisted of 284 new isolates from day 7 of adaptive mutation experiments and 16 isolates that were reported before [10]. These were included to determine whether there was complexity in the events that had not been detected by our previous method of outward PCR [10]. Indeed, two of the 16 carried an additional event identified by aCGH. We also studied 180 cultures derived from Lac^−^ cells taken from lactose minimal medium starvation plates on day 5 (this is equivalent to day seven Lac^+^ colonies, because point-mutant Lac^+^ take 2 days to form visible colonies). We also analyzed 60 day seven Lac^+^ point mutant colonies, and include these with the Lac^−^ isolates from starvation plates as a control of 240 stressed isolates that do not carry *lac*-amplification. In addition, we studied 60 single cell Lac^−^ colonies that had not been stressed. No change in copy number was found in any of 300 non-amplified samples.

All 300 unstable Lac^+^ isolates were found to carry amplification at *lac*. The mean copy number at *lac* was 69.3+/−22.9 (mean +/− SD). The mean length of 298 amplicons was 22.7 kilobase pairs (kb). These same isolates, when grown in lactose minimal medium (to maintain selection for amplification) were found to have about twice the amount of F′-borne chromosomal sequence than sequences that were only present on the chromosome (1.88+/−0.30-fold). There was no increase in copy number of F′ sequences in previously stressed Lac^−^ cells grown in glycerol minimal medium or in previously stressed Lac^+^ point mutant cells grown in lactose minimal medium (0.99+/−0.10-fold). We sequenced the amplification junctions of 40 amplicons. We found that all had microhomology at the junction sequence. Sixteen of the 40 were located in REP sequences [23]. The sequences of the 40 amplification junctions are shown in Table S1.

**Figure 1:**
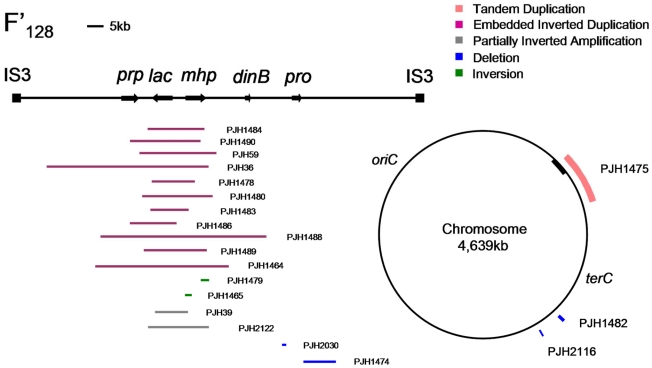
Distribution of structural changes in *E. coli* genome. A map of the chromosomal sequence on F′_128_ bounded by IS3 elements [41] and of the *E. coli* chromosome showing the positions of some of the structural changes reported here. The chromosomal origin of sequences carried on the F′ is shown as a black bar on the chromosome.

In 300 *lac*-amplified isolates, we identified 28 events that changed chromosomal structure in addition to the amplification at *lac* (9.3%). The positions of some of these changes on the standard map of *E. coli* are shown in Figure 1. The difference in the occurrence of other events in amplified isolates compared with zero in the 240 stressed control samples is highly significant (p = 0.0001; Fisher's exact test). Using the Peto Odds Ratio we can estimate the odds ratio (OR = 6.7) and a corresponding 95% confidence interval ranging from 3.1 to 14.1 [24]. Some of these additional events changed the copy number at *lac*, and might therefore have played a role in *lac*-amplification. Other events do not appear to offer a growth advantage to Lac^−^ cells on lactose minimal medium, and therefore represent other events occurring in the same cells as *lac*-amplification. We tested whether one inversion affected the rate of amplification by measuring amplification in a derivative that had lost amplification. Figure S2 shows that the inversion had no effect on rate.

### Events related to *lac*-amplification

**Figure 2:**
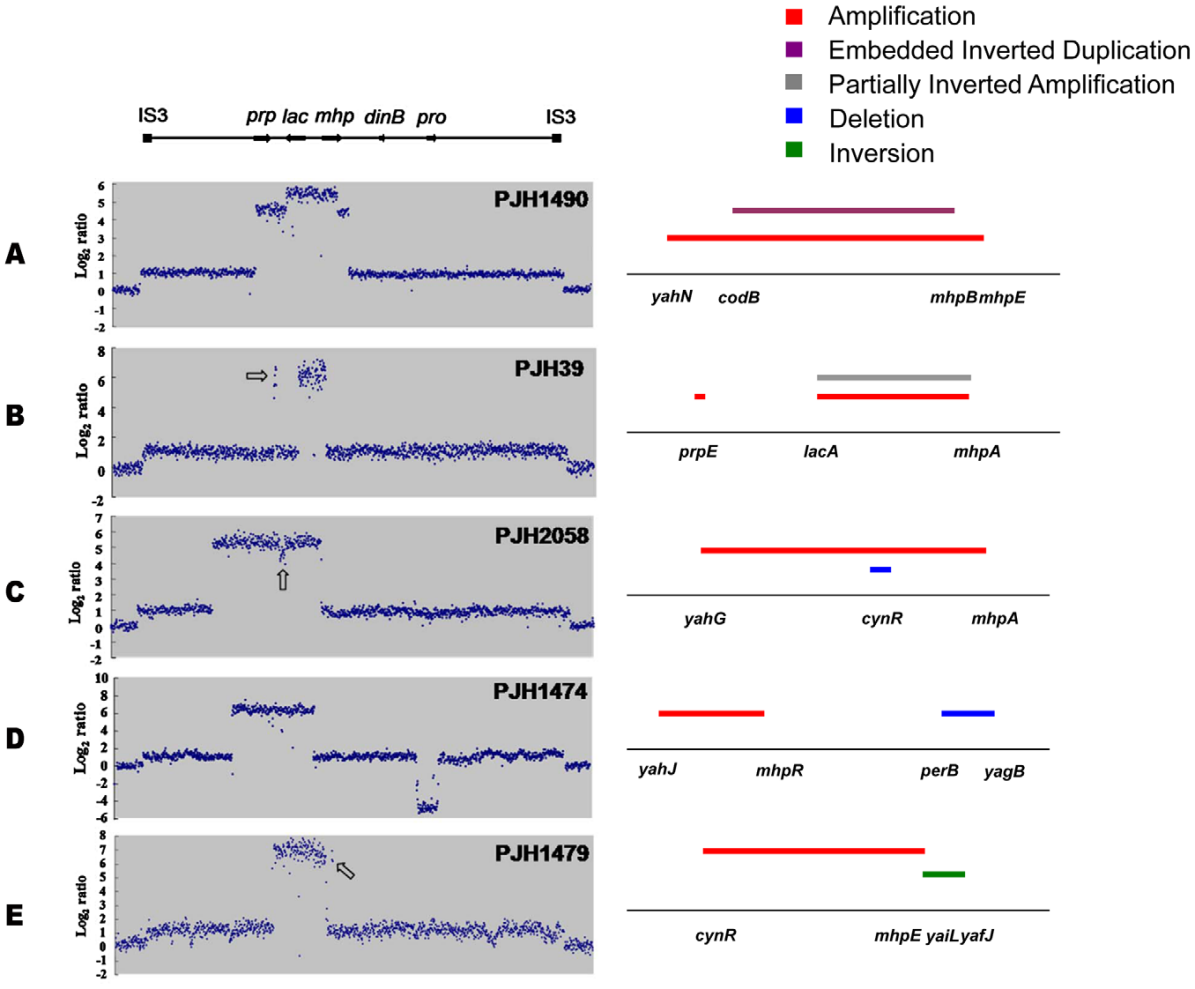
Complex rearrangements on the F′_128_ revealed by oligonucleotide aCGH and confirmed by PCR and DNA sequencing. a: Inverted region embedded in the amplification. b: Partially inverted amplification. c: Related deletion embedded in the amplification. d: Deletion independent of amplification. e: Inversion partially overlapping with amplification. Open arrows indicate features described in the text.

**Figure 3:**
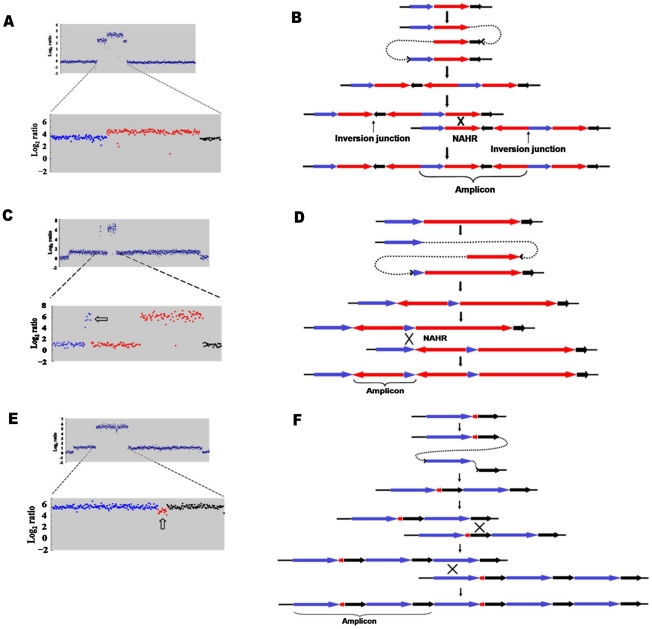
Model for the formation of complex structures by template switch events. The same model of repeated template switch can explain all these events, differing only in the position and orientation of the switch. a: Array CGH scan showing amplification with embedded inverted duplication. The blue, red and black dots correspond to blue, red and black arrows in b. b: Two inverted template switches that generate an inverted triplication (red) an inverted duplication (black) and a direct duplication, followed by unequal crossing-over (NAHR) between direct repeats (red or black) to generate the amplicon as observed. c: Array CGH scan showing partially inverted amplification. The blue, red and black dots correspond to blue, red and black arrows in d. d: Two inverted template switches generate an inverted duplication of part of the red sequence and a direct duplication part of the blue sequence. Unequal crossing-over can occur between blue sequences to generate the amplicon. e: Array CGH scan showing amplification consisting of a partially deleted duplication. The blue, red and black dots correspond to blue, red and black arrows in f. f: Two direct template switches give rise to an embedded deletion. Orientations were determined by sequencing the junctions (Table 1).

The most common complexity was an inverted duplication embedded in the amplified region (Figure 2a, PJH1490). This was found in 16 of 300 amplified isolates (5.3%) (Table 1). The same configuration was found to be common in the study by Kugelberg et al. with the Lac assay in *Salmonella enterica* [8]. In all 16 cases, the *lac* region was included in the embedded duplication. Detailed study of these events showed that the embedded inverted duplications vary in size from 5.2 to 42.6 kb. Two novel junctions were found in each case. The junctions showed microhomology of 3 to 30 bp (Table 1). We interpret these events as two inverted template switches that generate an inverted triplication, followed by unequal crossing-over that generates the amplified array (Figure 3, see Discussion).

**Table 1:** Sequences of junctions of complex events for those events for which there was more than one junction.

Strain	Size	Secondary SV	Tandem duplication junction	Amplification junction
PJH1475	301.6 kb	TD	IS*5* (*insH*)	TCAGG

			Amplicon and inversion junction	Inversion junction
PJH1484	13 kb	EID	CCGCC	AGCGACTC
PJH1478	10 kb	EID	TGG	TCCAGGCG
PJH1490	15.5 kb	EID	GCCGCC	GCCCAG
PJH1488	42.6 kb	EID	GCGGGCAG	GCGGCTACGAT
PJH1486	12.8 kb	EID	ACCGGTCAT	ACG
PJH2184	5.2 kb	EID	CTGG	GCTGGTG
PJH2052	25 kb	EID	GCAGCAGGC	ACCGTG
PJH2185	13.7 kb	EID	*GACGCGT*	CTC
PJH1489	17.3 kb	EID	*GACGCGT*	TCAGGTTGGC
PJH2134	17.1 kb	EID	*CGGCCTACGG*	TCAGGTTGGC
PJH36	37.8 kb	EID	GCTGCCGCGC	TTGGCGCGATT
PJH1464	24.3 kb	EID	GCTGCTGACCGA	GCCAACCTGA
PJH1489	17.5 kb	EID	AGCGTCGCATCAGGCATC	GCCAACCTGA
PJH1483	12 kb	EID	GTAGGCCTGATAAGACGCG	AAAACGGCGAG
PJH1480	17.1 kb	EID	GCGCACGACTGCCGGATGCGGCGT	GCCAGCCACAC
PJH59	18 kb	EID	*AGCGTCGCATCAGGCATCTGCGCACGACT*	*AGGACGTTG*
PJH39	7.9 kb	PIA	TGGCGGTGGCG	CGCCAGC
PJH2122	13.7 kb	PIA	CGCGTCTTATCA	GCCTGCT
PJH1465	1.5 kb	IN	CG	AGCGAT
PJH 1479	2.4 kb	IN	*TAGCGTCGCATCAGGCATCTGCGCACGACT*	*ATTTGTAGG*

			Deletion left junction	Deletion right junction
PJH1482	1.6 kb	DE	…GACGCTTTT**G**tataacacttca…	…cattagctaa**G**GAAGGTGCG…
PJH2116	0.8 kb	DE	…TTGCTGT**CTGC**tggttcagtaa…	…aagtttattg**CTGC**ACTTGTTG…
PJH2030	0.2 kb	DE	…GTGAATT**CTGG**ggctggtgg…	…aatctgaag**CTGG**AACCGGCG…
PJH1474	7.5 kb	DE	IS element	IS element
PJH2058	1.2 kb	EDE	…AAAA**CGTCGCGGCA**tcacg…	…tccaact**CGTCGCGGCA**AAGC…
PJH1477	23.8 kb	ND		
PJH1487	12.3 kb	ND		

TD: Tandem duplication; SV: Structural Variant; EID: Embedded Inverted Duplication; PIA: Partially Inverted Amplification; IN: Inversion; DE: Deletion; EDE: Embedded Deletion; ND: Not-identified; Sequences in italics are located inside REP sequences. Sequences in italics and underlined represent REP between *mhpE* and *mhpT*. For deletions, sequence deleted is shown in lower case, and junction sequences are bold. Array scans for PJH1475, PJH1477 and PJH1487 are shown in Figure S3.

We identified two other inverted regions that generated a distinct pattern on aCGH data where part of the amplicon appears to be detached from the rest on the map of the parental strain based on the standard map of *E. coli* (PJH39 and PJH2122) (one example, PJH39, is indicated in Figure 2b by an open arrow). When the map is corrected to include this inversion, the amplicon is seen to be contiguous. These events show only two novel junctions, the right end of the inversion and the amplification being the same junction. We therefore regard the inversion and the duplication as parts of the same event, and explain them below as a pair of inverted template switches followed by unequal crossing over (Figure 3c, 3d).

Another event of the same type, PJH2058, that did not involve inversion or duplication of *lac* (apart from the amplification) is shown in Figure 2c. There is a short sequence within amplicon that is present in 2-fold less copy number than the rest of the amplicon (open arrow in Figure 2c). This can also be explained by 2 switches, but neither of them is inverted (Figure 3e, 3f, see below).

A very large tandem duplication (about 300 kb) was found in an isolate (PJH1475) in which the F′-factor was integrated into the chromosome, so that part of the F′ including *lac*, and part of the chromosome was duplicated (Figure 1). We have confirmed the HFR status of this isolate by showing that conjugational transfer of *proAB*, which is on the F′-plasmid in FC40, is RecA-dependent in this isolate, whereas it would not be if it were situated on a plasmid. The duplication is flanked by IS*5* sequences, and therefore was presumably formed by homologous recombination (Table 1). Similarly, the integration of the F′-plasmid occurred by homologous recombination between sequences that are in common between the chromosome and F′_128_, because aCGH detected no other copy number change.

Two other large duplications, PJH1477 and PJH1487, were found that included *lac* and had one or both ends outside the chromosomal sequence on the F′. The junctions were not found in the IS*3* elements that span chromosomal sequence on the F′-plasmid as has been observed previously [8], [9]. The same two events contained duplications within the amplified segment. The junction sequences of both duplications were found to be recalcitrant to amplification by PCR. Multiple primer pairs were used in all pair-wise orientations, but no product or only unspecific product was found. Similar results have been reported for some human non-recurrent copy number changes (e.g. [25]). It is possible that these represent translocations, further unanticipated orientational complexities at the breakpoint junctions, or insertions of large genomic sequences/structures between the designed primers that do not correspond to a preconceived notion based on a reference genome sequence used for primer design. Array CGH provides copy number information, but neither positional nor orientational information. We were unable to characterize these further.

These data establish that, like in human, a significant proportion of events of chromosomal structural change that generate amplification are complex in that more than one structural change occurred, apparently within the same event. This applies to 19 of 300 events resolved by our approach (omitting large duplications that might have assisted amplification, but might not be part of the same event).

### Secondary events in *lac*-amplified isolates

In the same sample of 300 amplified isolates, we also found six that included a chromosomal structural change that was not apparently directly involved in the amplification. None was seen in the 240 stressed control isolates. The null hypothesis that the amount of that unrelated chromosomal structural change does not differ between amplified and stressed non-amplified isolates, can be rejected (p = 0.036; Fisher's exact test [26], [27]). Using the Peto Odds Ratio we can estimate the odds ratio (OR = 6.2) and a corresponding 95% confidence interval ranging from 1.2 to 31.0. [24].

Duplications should be unstable, so it is not surprising that we saw none that did not duplicate *lac* and thereby provide selection for maintenance of the duplication. Four of the unselected events were deletions (1.33% of 300 events): two on the F′-plasmid and two on the chromosome. One of the deletions (PJH1474) was flanked by non-identical IS elements, and so might have occurred by homeologous recombination or alternatively might have utilized the shorter homology stretches to mediate a template switch. The other three show microhomology junctions (1 to 4 bp), and so probably happened by events similar to those generating amplification. The chromosomal deletions were 0.8 and 1.6 kb long, and are situated at about 1.4 and 1.6 megabases on the standard reference *E. coli* map (PJH2116 and PJH1482 respectively). Deletions of 0.2 and 7.5 kb long (PJH2030 and PJH1482 respectively) were found on the F′ at about 44 kb and 50 kb from *lac* respectively (Figure 1). An example, PJH1474, is shown in Figure 2D.

**Figure 4:**
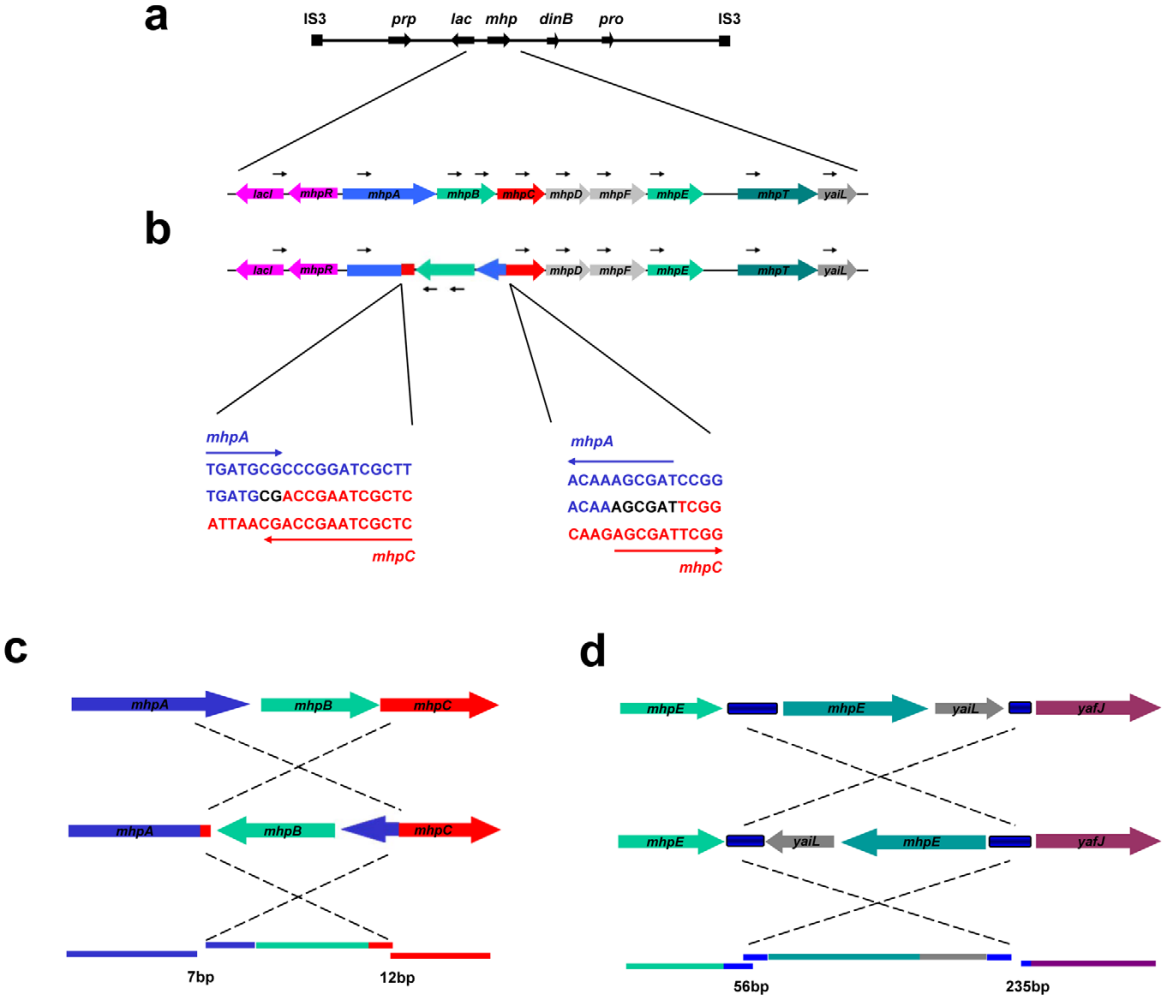
Inversion-associated small deletion and insertion mutations. a: Unidirectional PCR primers to the right of *lac* give no pcr product. b: when part of the sequence is inverted, a PCR product can be obtained. c: PJH1465; d: PJH1479. The inverted segment is shown in its original orientation to reveal the mutations at each end. When the inverted segments were compared with the original sequence, small deletions and insertions were found on both end points. Blue blocks between *mhpE* and *mhpT*, *yaiL* and *yafJ* are REP. Colored lines represent genes in the same colors as the colored dots. When the sequences are restored to their genomic positions, small duplications and deletions are revealed (lower parts of c and d). The lengths of micro-duplications and -deletion in base-pairs are given below the diagram.

We found one inversion because it made an apparent separation of the amplicon into two parts (based on the standard map) (Figure 2e, PJH1479). The endpoints of the inversion and the amplification are different, so we see no evidence that the events are related. The inversion presumably happened before the amplification, and the amplification then included part of the inverted region. Because most inversions would not be detected by aCGH, we searched all 300 amplified and 240 stressed control isolates for inversion within 20 kb to either side of *lac* by unidirectional PCR (Figure 4). When PCR primers point in the same direction, there is no PCR product unless the sequence at one of the primer binding sites has been inverted. We found one further inversion in an amplified isolate (PJH1465) and none in the controls. These two inversions are described in Figure 4. It is interesting that, although the exchanges were almost reciprocal, the junctions are not exactly in the same position, so that a mutation of a small deletion or insertion is made at either end of both inversions.

## Discussion

### Stress-induction of amplification

Kugelberg et al [8], [9], studying the Lac assay in *Salmonella enterica*, have proposed that amplification at *lac* is not induced by the stress of starvation, but is a product of selection for more β-galactosidase expression with parameters within those established for chromosomal structural changes in growing cells of *E. coli*. We regard these amplification events as stress-induced because it was not pre-existing [17] and has been shown to require two stress response regulators: the general and stationary-phase stress-response regulator σS (RpoS) [28], [29] and the periplasmic misfolded protein stress-response regulator σE (RpoE) [30]. The strong requirement for σS would appear to be definitive, except that a few RpoS-controlled functions are expressed in growing cells [31], so one might argue that it is growth-dependent functions that are required. This idea is refuted by the demonstration that the growth phase level of expression of σS is insufficient for adaptive mutation [14]. The strong requirement for the RpoE stress-response is for both formation and maintenance of amplification [30]. The requirement for two of the cell's major stress-response regulators is a strong argument for stress-induction of amplification.

### Complexity of events

We report that a significant number of amplification events are complex in that they show more than one novel junction, indicating more than one non-homologous recombination event. The case that amplification events in the Lac assay reflect template switches during replication has been made in detail elsewhere [6], [7]. The events described here are readily interpretable in terms of template-switching mechanisms, and support the concept. Figure 3 describes the template switch processes that we propose to have occurred to explain the complex events that we see, based on either the long-distance template switch model [10] or the MMBIR model [6]. Figure 3a and 3b show how two inverted template switches form an inverted triplication interspersed with direct and inverted duplications. Non-allelic homologous recombination (or unequal crossing-over) between directly duplicated regions will generate the complex amplicon that we see. Kugelberg et al. [8] use a very similar pattern of events to explain this configuration, which was also common in their data for amplification in *S. enterica*. Figure 3c and 3d shows how a different configuration, amplification overlapping an inverted region, which we saw twice (Figure 2b, PJH39 and PJH2122), can be derived very similarly from two inverted template switches followed by unequal crossing-over. The difference is only in the relative positions of the two template switches.

If an inverted template switch occurs, the product will not be a viable Lac^+^ clone under the conditions of these experiments unless there is a second inverted template switch. This is because a single inverted switch will generate an incomplete F′-plasmid. However, this requirement for a second inversion cannot be the explanation for all complexity because, as shown in Figure 3e and 3f, we also see evidence of double template switches in direct orientation, where no consideration of viability exists. In the case portrayed in Figure 3e, PJH2058, the amplicon consists of a direct duplication that has a deletion between the repeats. The events depicted in Figure 3e and 3f differ from those interpreted above that include inversions only in the positions and orientations of the switches.

### Chromosomal structural instability in a subpopulation

To determine whether a secondary structural change might play a role in the amplification process, we screened for loss of amplification in one strain carrying a secondary inversion. When this strain was used in a starvation-induced mutation experiment, the rate of amplification was unchanged from the control strain FC40 (Figure S2) suggesting that there is no functional reason for the occurrence of this inversion in a strain that carries amplification at *lac*. Taking the four deletions and the two inversions that did not share a junction with amplification as events secondary to amplification, we see a significant occurrence of secondary events in cells that underwent amplification compared with starved cells not showing amplification (p = 0.036; Fisher's exact test). This is clearly an under-estimate of structural changes because duplications would be expected to be unstable, so it is not surprising that we saw none that did not duplicate *lac*. Those duplications that include *lac* presumably provide selection for maintenance of the duplication [8], [9]. We only found inversions that were close to *lac* because we did not look elsewhere. Using aCGH, we would detect only those unrelated inversions that overlapped the amplicon, and we found one of these. We also looked for inversion by unidirectional PCR, but only in the 40 kb surrounding the *lac* locus.

The meaning of the finding that the sample of amplified isolates differs in the frequency of secondary events from non-amplified cells from the same plate is important. First, it means that amplifying cells differ from other cells in their propensity to undergo chromosomal structural change. Second, it shows that this happened in a subpopulation of starved cells rather than in the whole population of starved cells, because starved cells that did not undergo amplification provide the basis of comparison. The identification of chromosomal structural mutations that are secondary to the selected event is analogous to the finding in the Lac assay that *lac* ^+^ point mutation is correlated with an elevated frequency of other unselected secondary point mutations [18], [19], [20]. Third, the discovery in the same cells of events that are apparently separate from the amplification events shows that the structural changes occurring during starvation on lactose medium are not targeted specifically or exclusively to the *lac* locus.

The existence of this chromosomally unstable subpopulation is compatible with the concept of stress-induced differentiation to a condition permissive for chromosomal structural change and genomic instability, and is incompatible with models that seek to explain these events as normal change and selection in slowly growing cells. We suggest that this subpopulation is differentiated to a physiological condition that allows chromosomal structural change. This is suggested by the finding that some of the secondary events occurred on the chromosome, indicating that a diffusible factor is involved. Further, we suggest that this differentiation was induced by the stress of starvation.

### Mechanism of inversion

**Figure 5:**
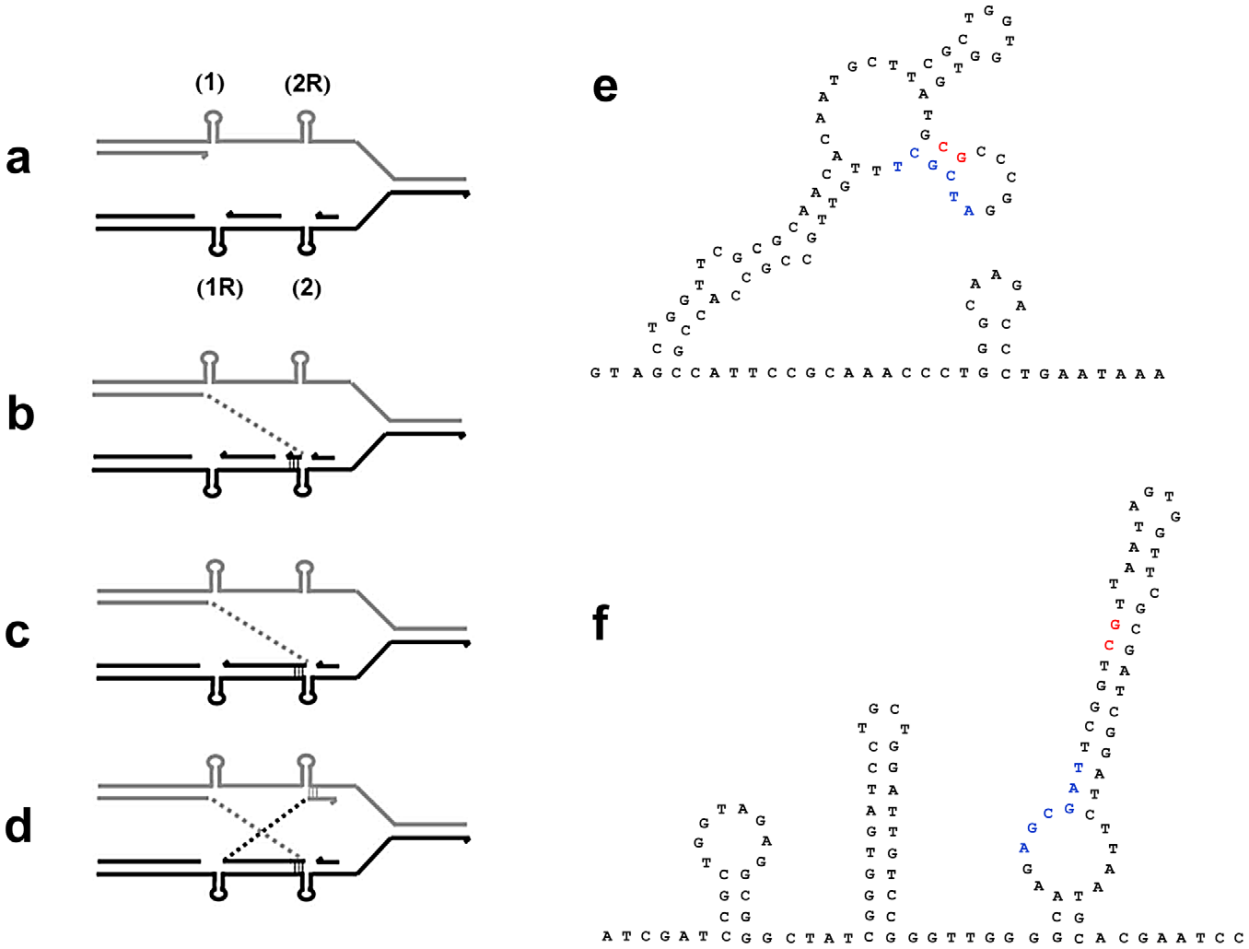
A model for inversion formation by template-switching giving non-homologous recombination in close to reciprocal positions. a: Leading-strand DNA synthesis is blocked by a secondary structure (1) in the leading-strand template. b: The stalled 3′-end bypasses blockage by an inverted switch to the lagging-strand template where lagging-strand synthesis has been blocked by a different secondary structure (2). c: Inverted DNA synthesis in lagging-strand stalled for the second time by the reciprocal secondary structure (1R). d: The stalled 3′-end switches to different reciprocal secondary structure (2R) in the leading-strand template. This allows replication to resume. e and f show potential secondary structure near the junctions in *mhpA* and *mhpC* respectively of the inversion in PJH1465. Left junction sequence is shown in red, right in blue.

We made the unexpected finding that some inversions involve almost, but not quite, reciprocal non-homologous recombination so that the junctions show insertions or deletions of a few tens of base-pairs. We suggest that this might occur as follows: If a template switch occurred because a replication fork was stalled by secondary structure forming in template DNA, then the complementary sequence on the other template would also be capable of forming a similar secondary structure. If two such sites occurred in a short interval, within the dimensions of a single replication fork, then the series of template switches portrayed in Figure 5 might explain how the almost reciprocal recombination occurred to form the inversion. Uncoupling of lagging-strand synthesis, after leading-strand synthesis is stalled by secondary structure (labeled “1” in Figure 5), might allow both structures to form on both strands, and so expose one to two kb of single-stranded sequence within the same replication fork. An inverted switch of the nascent leading strand from “1” to where lagging-strand synthesis is blocked ahead of the second secondary structure “2” is followed by synthesis in inverted orientation as far as the complementary secondary structure to the first blockage “1R”. The second template switch is to downstream of the complement to the second structure “2R”, thus completing the not quite reciprocal exchanges that flank the inversion. This allows replication to escape the blockage imposed by secondary structures. Figure 5 shows the secondary structures that could form in the regions involved in one of the inversions. All four junction sequences are in positions that can form a stem or a stem/loop of secondary structure. We suggest that at least these two inversion events formed by template switches [32], [33] within a replication fork induced by secondary structures in DNA.

### The role of REP

REP is a pseudopalindromic sequence of about 38 bp that occurs in clusters in intergenic regions [34]. Kugelberg et al. have noted that there is a tendency for junctions to occur at REP sequences [8], [9], and interpret this as evidence of homologous recombination. We found that 22 of 90 sequenced novel junctions (24%) occur at REP sequences (Table 1 and Table S1). Of these, 14 are too short for homologous recombination (5 to 20 bp) and 8 are in a range that might or might not allow homologous recombination (29 to 32 bp) [35], [36], but could also allow microhomology-mediated template switches as has been proposed for *Alu* repetitive sequences in the human genome [5]. REP clusters are rich in potential to form secondary structures. Figure S1 shows secondary structure predicted in a cluster of REP sequences near *lac* that is involved in 20% of the junctions listed in Table 1 and Table S1. We suggest that the propensity of the region to form secondary structures, rather than homology, is instrumental in forming this hotspot.

Study of the positions of novel junctions of amplification show a preference for the stem of potential stem-loop structures. For 40 amplification junctions that we sequenced (Table S1), 16 are REP sequences, and therefore rich in potential secondary structures. Analyzing potential secondary structures in the regions close to the junctions of the other 24 amplicons, only one junction sequence is confined to predicted unstructured sequence, and only one is confined to a potential hairpin loop. Those in the commonest class (10/24) occur on the stems of predicted secondary structures, and 9/24 are on both a stem and a loop. We considered that secondary structure might target amplification by blocking the progress of replication forks, or it might function to provide single-stranded DNA to which a primer could anneal during template switching. Because a minority of junctions are situated on a predicted hairpin loop where single-stranded DNA might occur, we favor the hypothesis that secondary structures target amplification by blocking replication. Others have reported that secondary structures in DNA are involved in chromosomal structural change [37]. Direct evidence of fork stalling at inverted repeats *in vivo* strongly suggests that stalling is mediated by hairpin formation on the lagging-strand template at replication forks [38].

### Mechanism of copy number change at *lac*

We have suggested above that some inversions are formed by template switching within a replication fork. Template switching is much more difficult to apply to other events reported here because most switches cover tens of kb, well beyond the 1.5 kb dimensions of a replication fork in *E. coli*. For this reason, we suggested previously that template switches occur between different replication forks: the long distance template-switch model [10]. Based on the evidence that double-strand breaks are involved in amplification at *lac*, we later suggested that the mechanism was a modification of break-induced replication (BIR) at collapsed replication forks, namely that in place of RecA-mediated strand invasion, the broken end annealed by microhomology to nearby single-stranded DNA (MMBIR) [6]. We know from experiments that use I-*Sce*I endonuclease to make double-strand cuts near to *lac* that double-strand breaks increase amplification at *lac* [14]. From this we suggested that nicking at *oriT* by TraI provides a discontinuity in the DNA template that leads to replication fork collapse [6] followed by MMBIR.

### Conclusion

We present evidence that, among stressed cells, a small proportion enters a state of heightened genomic instability during which multiple chromosomal structural changes might occur anywhere in the genome. Many such changes would be expected to be disadvantageous, but rarely a change occurs that allows escape from the stress. Because the events that we studied here in a bacterial model system are similar to those described for copy number changes in human, this conclusion might apply generally throughout biology. This view suggests that genome evolution might occur in bursts of multiple simultaneous chromosomal changes induced by stress. This view also has implications for understanding cancer progression in the stressful tumor microenvironment and the stresses imposed by chemotherapy, both of which might induce showers of chromosomal structural changes.

## Materials and Methods

### Strains

*Escherichia coli* cells of strain SMR4562 [39], isogenic with FC40 [16] carry the conjugative plasmid F′_128_ with a leaky *lac +*1 frameshift mutation were initially grown to stationary phase for 3 days at 32° [14]. We then followed the standard procedure [40] for adaptive mutation experiment in the Lac assay [16]. Lac^+^ colonies arise over several days, and are marked daily. Amplification was distinguished from point mutation by its instability as seen by blue and white sectoring of colonies grown on rich medium with X-gal. 284 Lac^+^ colonies from day 7 together with 16 previously published amplified strains [10] were collected for further study. We also studied 60 Lac^+^ colonies arising on day 7 that carried point mutations reverting the *lac* mutation. 180 Lac^−^ stressed FC40 control cells were collected by taking plugs from the same lactose plate on day 5. Sixty colonies derived from unstressed control cells were taken from the initial stationary phase culture.

These 584 new isolates are identified by strain numbers PJH1458–PJH1642 and PJH2025–PJH2425. Those described previously [10] are strains PJH2, PJH5, PJH6, PJH7, PJH19, PJH20, PJH22, PJH26, PJH27, PJH39, PJH59, PJH64, PJH79, PJH80, PJH81 and PJH165.

### Array comparative genomic hybridization (aCGH)

Total genomic DNA was extracted from exponential culture in M9 lactose medium for Lac^+^ isolates or M9 glycerol medium for Lac^−^ isolates by using the QIAGEN DNA Purification kit. *E.coli* custom high-resolution genomic microarray (4×44K) containing 44,000 unique sequence oligonucleotides spaced at about 100-bp intervals were obtained from Oxford Gene Technology (OGT). Probe labeling and hybridization were performed following the manufacturer's protocol (Agilent Oligonucleotide Array-based CGH for Genomic DNA Analysis). Slides were scanned on a GenePix 4000B Microarray Scanner (Axon Instruments). Data extraction, normalization and visualization were achieved by using Agilent Feature Extraction Software A.7.5.1. Extraction data were analyzed for copy number differences by using Microsoft Excel software. All occurrences of two or more adjacent probes showing 2-fold or more increase or decrease in copy number relative to the reference FC40 DNA were investigated further, except those that mapped to repetitive elements or prophages.

### Structure confirmation by PCR and sequence analysis

All deletion, inversion and duplication junctions were further validated by PCR and sequencing. Inward-facing primers for deletions and inversions and outward-facing primers for tandem duplication were designed based on sequence from National Center for Biotechnology Information (NCBI) *Escherichia coli* K-12 *substr*. MG1655. Long-range PCR was performed using LongAmp™ *Taq* Master Mix (New England Biolabs). The PCR products were purified with either a QIAquick PCR Purification Kit (QIAGEN) or a QIAEX II Gel Extraction Kit (QIAGEN) following the manufacturer's instructions, and sequenced by Lone Star Labs (Houston, Texas, United States). DNA sequences were analyzed by comparison to reference sequences with the use of BLAST (http://blast.ncbi.nlm.nih.gov/Blast.cgi). Possible secondary structures in DNA were found by use of DNAMAN version 6 (Lynnon Biosoft).

### Deamplification

Deamplified lines were derived from amplified isolates by screening for sectors in colonies of amplified strains that showed a low level of β-galactosidase as seen on medium containing X-gal, but yet retained the ability to grow without proline.

## Supporting Information

Figure S1 Potential second structure of an array of REP sequences between *mhpE* and *mhpT*. Amplification junctions that were identified in this region are shown in red.
(DOC)

Figure S2 Inversion has no effect on amplification. A deamplified derivative strain of PJH1465 shows the same rate of amplification as the wild-type FC40. Mean with SEM of four cultures. Diamonds: FC40. Squares: deamplified PJH1465.
(DOC)

Figure S3 Array scans for PJH1475, PJH1477 and PJH1487.
(PPT)

Table S1 Sequences of 40 amplicon junctions.
(DOC)
