# Supplementary material for: Global Chromosomal Structural Instability in a Subpopulation of Starving Escherichia coli Cells
Source: PLoS Genet. 2011 Aug 25;7(8):e1002223. doi: 10.1371/journal.pgen.1002223 (PMC3161906; doi:10.1371/journal.pgen.1002223)
Supplement: Figure S1 — Potential second structure of an array of REP sequences between mhpE and mhpT. Amplification junctions that were identified in this region are shown in red. (DOC) [file pgen.1002223.s001.doc]

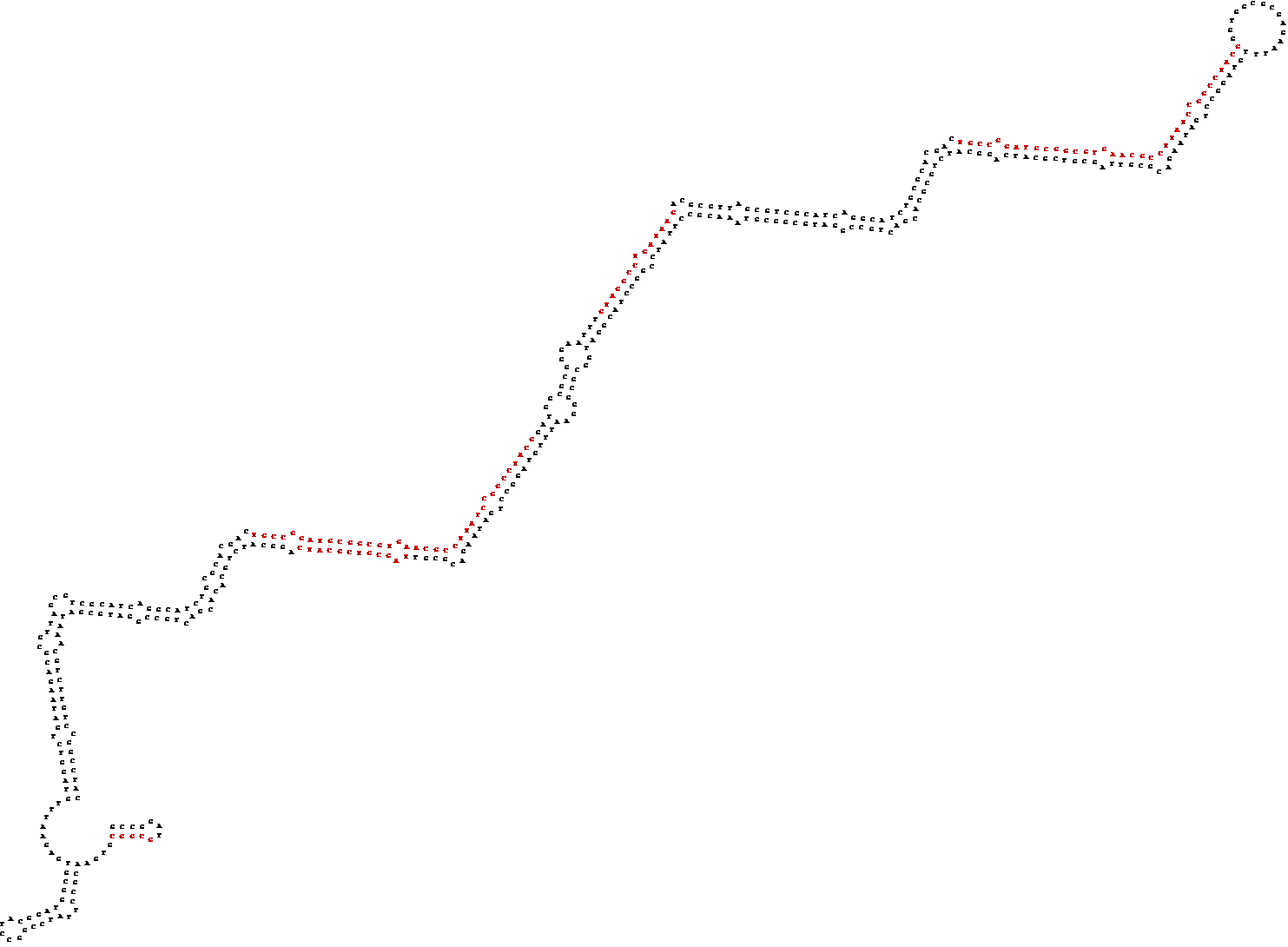


**Figure S1.** Potential second structure of an array of REP sequences between *mhpE* and *mhpT.*  Amplification junctions that were identified in this region are shown in red.
