# Supplementary material for: Global Chromosomal Structural Instability in a Subpopulation of Starving Escherichia coli Cells
Source: PLoS Genet. 2011 Aug 25;7(8):e1002223. doi: 10.1371/journal.pgen.1002223 (PMC3161906; doi:10.1371/journal.pgen.1002223)
Supplement: Figure S2 — Inversion has no effect on amplification. A deamplified derivative strain of PJH1465 shows the same rate of amplification as the wild-type FC40. Mean with SEM of four cultures. Diamonds: FC40. Squares: deamplified PJH1465. (DOC) [file pgen.1002223.s002.doc]

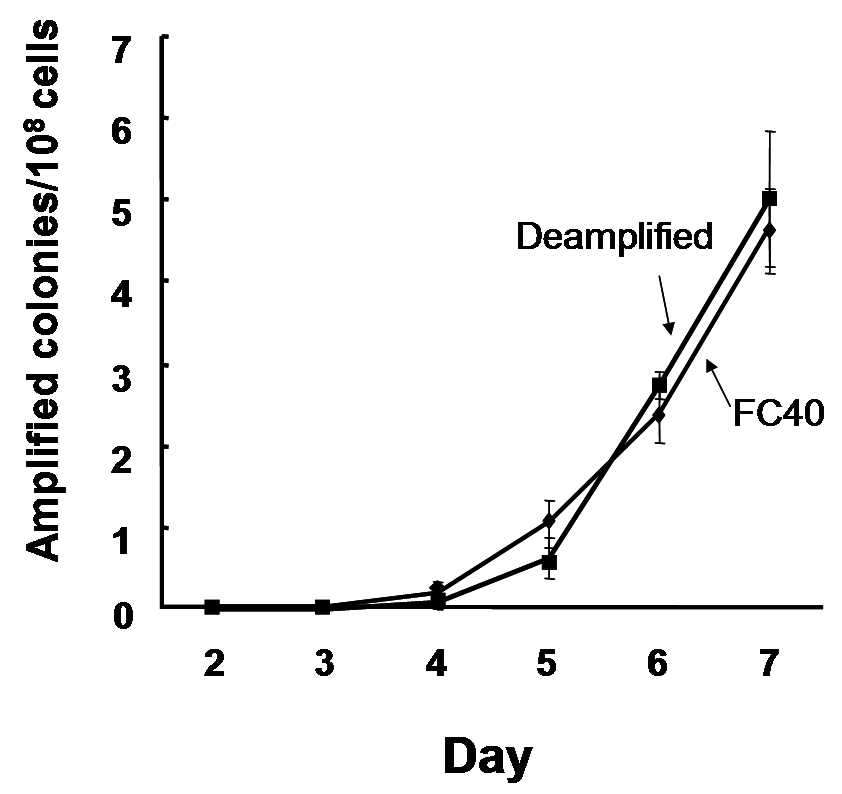


**Figure S2.** Inversion has no effect on amplification. A deamplified derivative strain of PJH1465 shows the same rate of amplification as the wild-type FC40. Mean with SEM of four cultures. Diamonds: SMR4562. Squares: deamplified PJH1465.
