# Supplementary figures and images for: Global Chromosomal Structural Instability in a Subpopulation of Starving Escherichia coli Cells
Source: PLoS Genet. 2011 Aug 25;7(8):e1002223. doi: 10.1371/journal.pgen.1002223 (PMC3161906; doi:10.1371/journal.pgen.1002223)

## Slide 1
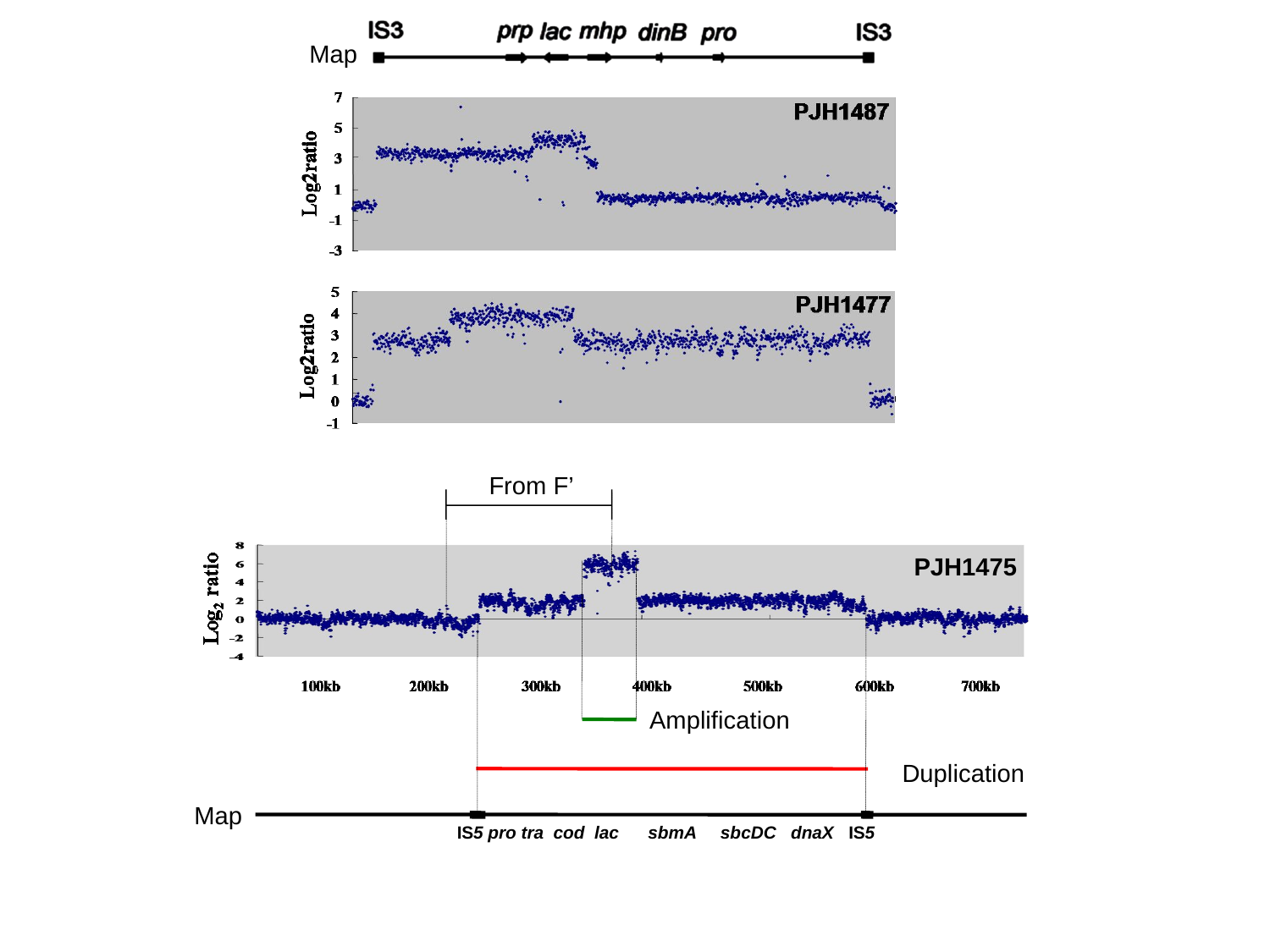

Map
From F’
PJH1475
Amplification
Duplication
Map
IS5 pro tra cod lac sbmA sbcDC dnaX IS5

Supplement: Figure S3 — Array scans for PJH1475, PJH1477 and PJH1487. (PPT) [file pgen.1002223.s003.ppt]
