# Supplementary material for: Global Chromosomal Structural Instability in a Subpopulation of Starving Escherichia coli Cells
Source: PLoS Genet. 2011 Aug 25;7(8):e1002223. doi: 10.1371/journal.pgen.1002223 (PMC3161906; doi:10.1371/journal.pgen.1002223)
Supplement: Table S1 — Sequences of 40 amplicon junctions. (DOC) [file pgen.1002223.s004.doc]

**Table S1. Sequences of 40 amplicon junctions.**

| Strains | Junction sequences | Location on chr. (amplicon left) | Location on chr. (amplicon right) |
| --- | --- | --- | --- |
| PJH2139 | GCGCG | 373403 | 356285 |
| PJH2112 | TAGCGTCGCATC | 374539 | 349091 |
| PJH2108 | ACTGCTGGCG | 368713 | 349649 |
| PJH2105 | CGTAGGCCGGATAAGGCGTTCACGCCGCATCCGGCA | 374393 | 357003 |
| PJH2102 | GCGTTTG | 370356 | 358987 |
| PJH2131 | TGCCGGATGCGGCGTGAACGCCTTATCCGGCCTACG | 374280 | 356707 |
| PJH2172 | CGTTTGCCA | 375189 | 355707 |
| PJH2095 | TTCGCC | 369854 | 355016 |
| PJH2142 | CGCGCC | 373643 | 352809 |
| PJH2145 | CCGCTGA | 370162 | 354777 |
| PJH2144 | CGTAGGCCGGATAAGGCGTT | 374443 | 356917 |
| PJH2086 | GGCAAACATC | 375456 | 354487 |
| PJH2162 | GGCGCGCGGGT | 373408 | 352590 |
| PJH2140 | GCGGCGGTGG | 373731 | 359616 |
| PJH2094 | CGCG | 376585 | 348841 |
| PJH2111 | CGTTTGCCATGC | 375189 | 355707 |
| PJH2159 | TGCCGGATGCGGCGTGAACGCCTTATCCGGCCTACG | 374280 | 356707 |
| PJH2117 | CCGGATAAG | 376615 | 244129 |
| PJH2114 | GCTGGTGG | 367964 | 353621 |
| PJH2137 | GTGACGG | 368358 | 358079 |
| PJH2127 | CGCGCGTT | 369231 | 356284 |
| PJH2131 | TGCCGGATGCGGCGTGAACGCCTTATCCGGCCTACG | 374280 | 356707 |
| PJH2136 | AGCGGCA | 373092 | 360254 |
| PJH2120 | GTTCCGGTGGGC | 370029 | 349306 |
| PJH2164 | GGTCGC | 370342 | 353326 |
| PJH2155 | TGCCTGGG | 371943 | 354632 |
| PJH2165 | ATGCCTGATGCGACGCT | 376664 | 353854 |
| PJH2156 | CGTAGGCCGGATAAGGCGTTCACGCCGCATCCGGCA | 374188 | 356907 |
| PJH2176 | TGCCGGATGCGGC | 374266 | 360399 |
| PJH2177 | GCGGC | 374165 | 357501 |
| PJH2178 | CGTTTGCCATGC | 375189 | 355707 |
| PJH2180 | GATCCCGC | 351497 | 261324 |
| PJH2169 | AACCCGATTT | 370209 | 355340 |
| PJH2158 | TGCCGGATGCGGC | 374472 | 360399 |
| PJH2147 | CCGCCGCCGTTT | 373989 | 351373 |
| PJH2150 | CTTATCAGGCCTAC | 374420 | 356955 |
| PJH2147 | TGCCGGATGCGGCGTGAACGCCTTATCCGGCCTACG | 374188 | 356907 |
| PJH2184 | CTTATC | 374185 | 349048 |
| PJH2168 | CAACTGAGC | 374948 | 358482 |
| PJH2181 | GGCAACTCG | 370804 | 354848 |

Red and blue colors represent different REP sequences (red: REP sequences between *mhpE* and *mhpT*)
